# Construction, expression, and characterization of AG1^1–843^ and AG1^1–1581^

**DOI:** 10.1016/j.dib.2018.08.094

**Published:** 2018-08-30

**Authors:** Xie Yan, Yan-Tao Yang, Wei Shi, Xia Ai, Xu-Guang Xi

**Affiliations:** a College of Life Sciences, Northwest A&F University, Yangling, Shaanxi 712100, China; b Laboratoire de Biologie et Pharmacologie Appliquée, Ecole Normals Supérieure de Cachan, CNRS, 61 Avenue du Président Wilson, 94235 Cachan, France

**Keywords:** Gene synthesis, Plasmid construction, PCR, Recombinant protein expression, DUF1220, AG1

## Abstract

This data article contains descriptive and experimental data on the construction, expression, and simple characterization of AG1^1–843^ and AG1^1–1581^. AG1 is an important member of the DUF1220 protein family. It׳s hard to get the recombinant protein because of its DNA sequence. The DNA sequence were optimized by proper design, cloned by overlap PCR and constructed into expression vector. AG1^1–843^ and AG1^1–1581^.were over expressed in *Escherichia coli*, purified and analyzed by dynamic light scattering and gel filtration analysis. An effective technique is provided to construct and express proteins with complicated sequences.

**Specifications Table**

**Table:** 

Subject area	biology
More specific subject area	Molecular biology, protein science
Type of data	Table, graph, figure
How data was acquired	EMSA, SDS-PAGE,
	Dynamic light scattering (DynaPro NanoStar instrument, Wyatt Technology Corporation, USA)
	Size exclusion chromatography (ÄKTA Purifier, GE Healthcare, USA)
Data format	Raw and analyzed
Experimental factors	none
Experimental features	DLS, gel filtration
Data source location	College of Life Sciences, Northwest A&F University, Yangling, Shaanxi 712100, China
Data accessibility	The data are available with the article.
Related research article	Construction, expression, and characterization of AG1^1–843^ and AG1^1–1581^.

**Value of the data**

• A method of amino acid and DNA sequence optimization, synthesis, recombinant protein expression for proteins with complicated sequences is provided.
• Sequence analysis and synonymous codon substitution was used for sequence optimization.
• Overlap-PCR was used for the sequence synthesis.
• The recombinant proteins AG1^1–843^ and AG1^1–1581^ were expressed and purified for further analysis.
• The existence state in solution of AG1^1-843^ and AG1^1-1581^ were analyzed by DLS and gel filtration.

## 1 Data

**Fig. 1:**
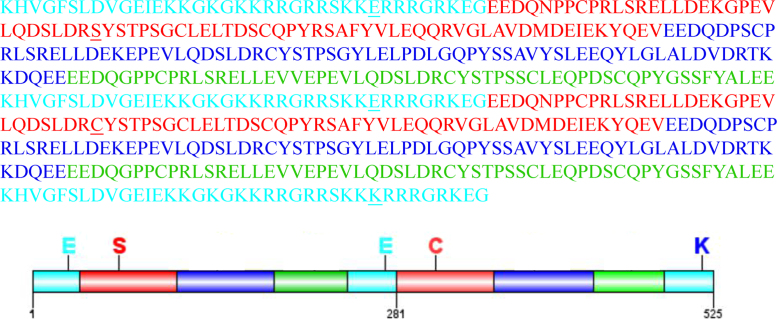
AG1 amino acid sequence comparison. Internal highly conserved sequence repeats are highlighted for each group. Cyan residues: 1–38, 245–281, and 489–525 (CRSA), which share 100% identity; Red residues: 38–112 and 282–356 (CRS1), which share 99% identity; Blue residues: 113–187 and 357–431 (CRS2), which share 100% identity; Green residues: 188–244 and 432–488 (CRS3), which share 100% identity. Furthermore, CRS1 and CRS2 share 75.7% identity, CRS2 and CRS3 share 58.7% identity, and CRS1 and CRS3 share 61.3% identity.

**Fig. 2:**
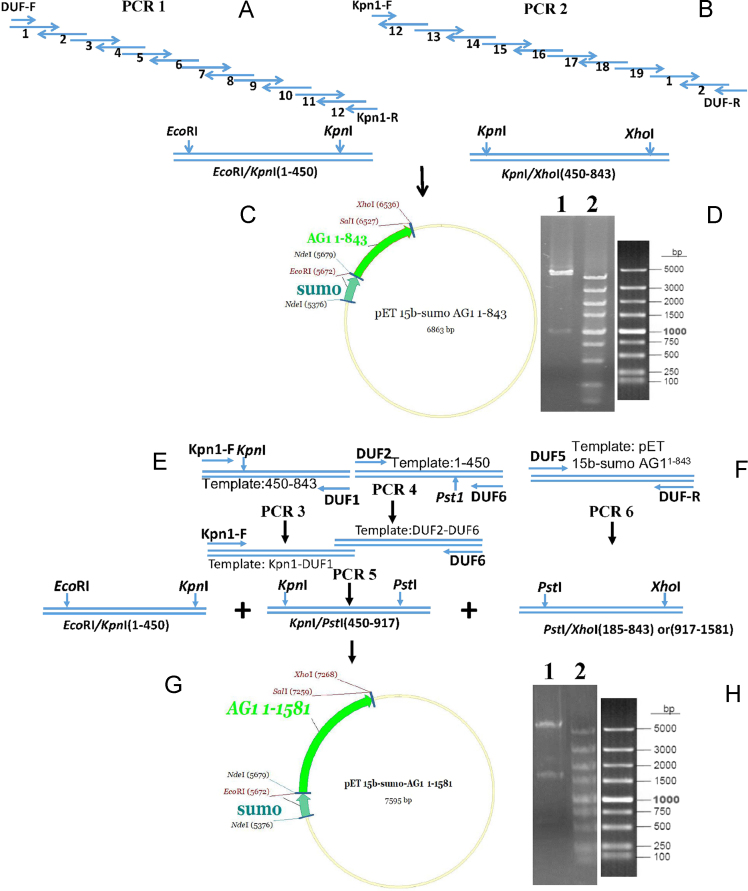
The construction process used for the pET-15b-sumo-AG1^1–843^ and pET-15b-sumo-AG1^1–1581^ vectors. (A) The strategy for the synthesis and assembly of the AG1^1–450^ fragment using overlap extension PCR. The AG1^1–450^ PCR product was cloned into pMD19-T (Fig. S1A). (B) The strategy for the synthesis and assembly of the AG1^450–843^ fragment using overlap extension PCR, followed by cloning of the fragment into pMD19-T (Fig. S1B). (C) The AG1^1–450^ and AG1^450–843^ fragments were mixed with pET-15b-sumo vector and sealed together via ligation of their restriction sites to form the recombinant expression plasmid (pET-15b-sumo-AG1^1–843^). (D) Enzymatic digestion of the recombinant vector (electrophoresed on a 1% w/v agarose gel). Lane 1: pET-15b-sumo-AG1^1–843^ digested with *Eco*RI and *Xho*I; Lane 2: 5000 bp DNA size marker. (E) The AG1^450–843^ and AG1^1–450^ fragments were used to assemble the AG1^1–843^ fragment in the third, fourth, and fifth PCR reactions. (F) The pET-15b-sumo-AG1^1–843^ plasmid was used to assemble the template for the sixth PCR reaction, obtaining the AG1^917–1581^ and AG1^185–843^ fragments. (G) The AG1^1–450^, AG1^450–917^, and AG1^917-1581^ were mixed with pET-15b-sumo vector and sealed via ligation of their restriction sites, to form the recombinant plasmid (pET-15b-sumo-AG1^1–1581^). (H) Enzymatic digestion of the recombinant vector. Lane 1: pET-15b-sumo-AG1^1–1581^ digested with *Eco*RI and *Xho*I; Lane 2: 5000 bp DNA size marker.

**Fig. 3:**
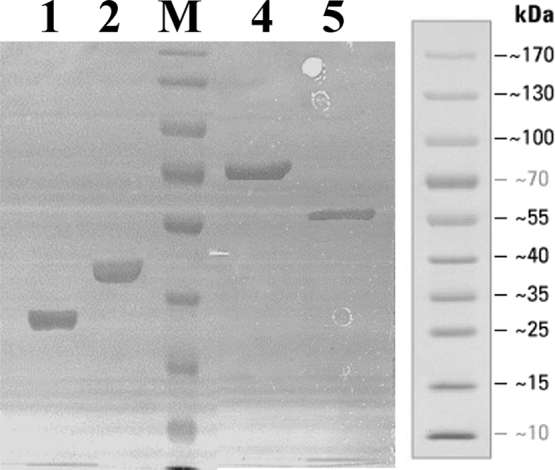
SDS-PAGE analysis of the purified AG1^1–843^ and AG1^1–1581^ recombinant proteins using a 10% polyacrylamide gel. 1: AG1^1–843^ recombinant protein; 2: undigested AG1^1–843^ recombinant protein containing sumo tag; 4: undigested AG1^1–1581^ recombinant protein containing sumo tag; 5: AG1^1–1581^ recombinant protein; M: standard protein size marker.

**Fig. 4:**
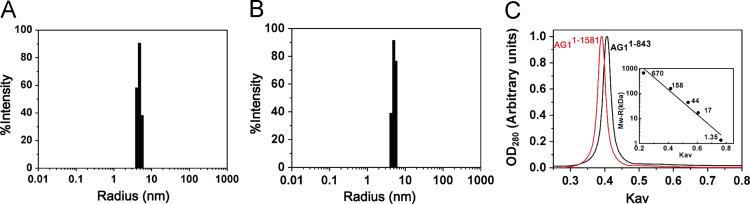
The quaternary structure of the recombinant AG1^1–843^ and AG1^1–1581^ proteins in solution. (A) Dynamic light scattering analysis of recombinant AG1^1–843^. (B) Dynamic light scattering analysis of recombinant AG1^1–1581^. (C) Size exclusion chromatography analysis of AG1^1–843^ and AG1^1–1581^ using a Superdex 200 10/300 GL column.

There were several repeat sequences in AG1 coding sequence which is the obstacle for cloning (Fig. 1). Synonymous codon substitution was used to optimize the sequence and then the sequences were cloned into expression vectors (Fig. 2, Table 1). The recombinant proteins were purified (Fig. 3) and analyzed by DLS and gel filtration (Fig. 4).

**Table 1:** List of synthesis primers for AG1^1–843^ and AG1^1–1581^ construction.

Primer name	Primer sequence 5′→3′
DUF1	AAACACGTTGGTTTCTCTCTGGACGTTGGTGAAATCGAGAAGAAAGGTAAAGGTAAGAA
DUF2	ACCACGACGACGTTCCTTCTTAGAACGACGACCACGACGCTTCTTACCTTTACCTTTCT
DUF3	GAAGGAACGTCGTCGTGGTCGTAAAGAAGGTGAAGAAGACCAGAACCCGCCGTGCCCGC
DUF4	ACTTCCGGACCCTTCTCGTCCAGCAGTTCACGAGACAGACGCGGGCACGGCGGGTTCTG
DUF5	ACGAGAAGGGTCCGGAAGTTCTGCAGGACTCTCTGGACCGTTCTTACTCTACCCCGTCT
DUF6	AACGGTACGGCTGGCAAGAGTCGGTCAGTTCCAGGCAACCAGACGGGGTAGAGTAAGAA
DUF7	TCTTGCCAGCCGTACCGTTCTGCGTTCTACGTTCTGGAACAGCAGCGTGTTGGTCTGGC
DUF8	TTCAACTTCCTGGTACTTCTCGATTTCGTCCATGTCAACCGCCAGACCAACACGCTGCT
DUF9	GAAGTACCAGGAAGTTGAAGAAGACCAGGACCCGTCTTGCCCACGCTTATCGCGCGAAT
DUF10	AGTGAATCTTGTAGCACCTCAGGCTCTTTTTCATCAAGCAATTCGCGCGATAAGCGTGG
DUF11	AGGTGCTACAAGATTCACTTGATCGGTGTTATTCAACACCCTCAGGATACCTGGAACTG
DUF12	GAGAGTAAACCGCAGAAGAGTACGGCTGACCCAGGTCCGGCAGTTCCAGGTATCCTGAG
DUF13	TCTTCTGCGGTTTACTCTCTGGAAGAACAGTACCTGGGTCTGGCGCTGGACGTTGACCG
DUF14	CGGACCCTGGTCTTCTTCTTCTTCCTGGTCCTTCTTGGTACGGTCAACGTCCAGCGCCA
DUF15	AAGAAGAAGACCAGGGTCCGCCATGCCCCAGGCTCAGCAGGGAGCTGCTGGAGGTAGTA
DUF16	AGGGAGCTGCTGGAGGTAGTAGAGCCTGAAGTCTTGCAGGACTCACTGGATAGATGTTA
DUF17	ACTCACTGGATAGATGTTATTCAACTCCTTCCAGTTGTCTTGAACAGCCTGACTCCT
DUF18	TCTTGAACAGCCTGACTCCTGCCAGCCCTATGGAAGTTCCTTTTATGCATTGGAGGAAA
DUF19	AGTTCCTTTTATGCATTGGAGGAAAAACACGTTGGTTTCTCTCTGGACGTTGGTGAAA
Kpn1-F	ATTCAACACCCTCAGGGTACCTGGAACTGCCGGA
Kpn1-R	TCCGGCAGTTCCAGGTACCCTGAGGGTGTTGAAT
DUF-F	GAATTCCATATGAAACACGTTGGTTTCTCTCTGGACGTT
DUF-R	GCCTCGAGTTAGTCGACACCTTCTTTACGACCACGACGACGTTCCTTCT

## 2 Experimental design, materials, and methods

### 2.1 Gene sequence analysis

The gene synthesis product in this study is that of the 1862 bp human AG1 gene (GenBank accession no: AF380580.1) which encodes the 615 amino acid DUF1220 AG1 protein fragment (http://www.uniprot.org/uniprot/Q8IX72). The AG1 gene and amino acid sequences are highly repetitive (Fig. 1). In order to increase the speed and efficiency of gene synthesis, we modified the AG1 nucleotide sequence. However, these changes did not affect the amino acid sequence. This codon optimization allows us to exploit the frequently used codons in *Escherichia coli* to obtain high level gene expression. Moreover, in order to improve the efficiency of gene transcription and RNA stability, the GC content of the synthetic gene was held at 52.9%. (Table 2, Table 3).

**Table 2:** Purify analysis of AG1^1–843^ and AG1^1–1581^.

Step	Protein name	Total protein^a^(mg)	Target protein(mg)	Purity^b^(%)	Yield(%)
Cell lysate^c^	AG1^1–843^	1012	–	–	–
	AG1^1–1581^	896	–	–	–
Supernatant	AG1^1–843^	209.2	–	–	100
	AG1^1–1581^	182.3	–	–	100
Ni^2+^ column elution	AG1^1–843^	24.6	20.7	84	9.9
	AG1^1–1581^	16.7	12.4	74	6.8
HiTrap Q elution	AG1^1–843^	15.5	14.6	94	7.0
	AG1^1–1581^	9.2	8.5	92	4.7
Final product	AG1^1–843^	14.8	14.4	97	6.9
	AG1^1–1581^	8.6	8.0	93	4.4

a Total protein was determined by NanoDrop ND-2000.

b Protein purity was estimated by SDS-PAGE image analysis.

c Lysate was obtained from cells of a 1.5 L culture.

**Table 3:** The characteristic constants of the AG1^1–843^ and AG1^1–1581^ recombinant proteins.

Analysis	AG1^1–843^	AG1^1–1581^
Length	281 aa	525 aa
Molecular	32,225.37	60,130.98
1 microgram	31.031 pMoles	16.630 pMoles
Molar Extinction coefficient	16,440	33,000
A A[280]corr.to	1.96 mg/ml	1.82 mg/ml
A[280]of 1 mg/ml	0.51 AU	0.55 AU
Isoelectric Point	5.19	4.87
Charge at pH 7	− 9.06	− 27.98

### 2.2 Oligonucleotide design and purification

The amino acid sequences of two human AG1 fragments (1–1581 bp, 1–527 aa and 1–843 bp, 1–281 aa) were obtained from GenBank (GenBank accession nos: AAO15403.1 and AAX85105.1). The amino acid and nucleotide sequences of these proteins were analyzed and codon-optimized using ClustalW 2.1, Vector NTI Viewer 4.0.1, Sequencher_v4.1., ExPASy Bioinformatics Resource Portal (http://www.expasy.org/), and NCBI Blast (https://blast.ncbi.nlm.nih.gov/Blast.cgi) software packages.

The design of the synthetic assembly oligonucleotides was similar to that of Xiong et al. [1], whereby each optimized DNA sequence was divided into economically sized oligonucleotides approximately 57–59 bases long that had 17–19 overlapping bases at both the 5′ and 3′ ends, leaving a 21 base gap between the overlapping regions. In addition, two outer amplification primers containing different restriction enzyme binding sites were designed for each gene to facilitate cloning. Both the AG1^1–843^ and AG1^1–1581^ sequences contained 23 oligoes. The oligonucleotides listed in Table 1 were from Sangon Biotech (Shanghai) Co., Ltd.

### 2.3 Rapid preparation of DUF1220 AG1^1–843^

We used the single overlap extension method as well as the two-step successive PCR method to synthesize the duplicated DUF1220 AG1 gene. Firstly, we mixed 14 (DUF1-DUF12, DUF-F, and Kpn1-R) and 12 (DUF13- DUF19, DUF-R, and Kpn1-F) chemically synthesized single stranded oligonucleotides (1 μM) in separate reaction tubes, followed by hybridization and extension to form the long dsDNA AG1^1–450^ (511 bp) and AG1^450–843^ (429 bp) constructs containing the appropriate restriction enzyme sites. AG1^1–450^ contained *Eco*RI and *Kpn*I sites, while AG1^450–843^ contained *Kpn*I and *Xho*I sites. The PCR reactions were conducted for 25 cycles with 5 U *Pfu* polymerase (NEB) in a final volume of 100 μl, in presence of 1× *Pfu* buffer and 200 μM dNTP. The PCR conditions were 10 s at 90 °C, 10 s at 60 °C, and 50 s at 72 °C for each cycle, followed by extension for 10 min at 72 °C, unless stated otherwise. The AG1^1–450^ and AG1^450–843^ gene fragments were then cloned into a simple pMD19-T vector and sequenced. The pMD19-T-AG1^1–450^ plasmid was then digested with *Eco*RI and *Kpn*I, while the pMD19-T-AG1^450–843^ was digested with Kpn 1 and *Xho*I, followed by separation on a 1% agarose gel. The digestion products (AG1^1–450^ and AG1^450–843^) were excised from the gel with a blade, and a purification kit (CoWin Biosciences) was used according to the manufacturer׳s instructions. Then, the AG1^1–450^ and AG1^450–843^gene fragments were cloned together into a pET-15b-sumo vector which contains 6× His tag and SUMO fusion tags. The molecular cloning of the synthesized DNA fragments was performed according to the standard procedures [1].

### 2.4 High efficiency preparation of DUF1220 AG1^1–1581^

Gene AG1^1–1581^ is composed of two repeats of the AG1^1–843^ fragment, meaning it can be built with the AG1^1–450^ and AG1^450–843^ fragments expressed in the pET-15b-sumo-AG1^1–843^ plasmid. Firstly, the AG1^450–843^ and AG1^1–450^ fragments and the pET-15b-sumo-AG1^1–843^ plasmid were used to assemble the template for the third, fourth, and sixth PCR reaction. The two outermost oligonucleotide primers used were Kpn1-F and DUF1, DUF2 and DUF6, and DUF5 and DUF-R, respectively. Secondly, the DNA segment from the Kpn1-DUF1 and DUF2-DUF6 reactions were mixed and used to assemble the template for the fifth PCR reaction, which was carried out using the Kpn1-F and DUF6 oligonucleotides as the two outermost primers. All of the PCR reactions used 5 U *Pfu* polymerase and 200 μM dNTP and were performed with the following program: 98 °C for 1 min, then 25 cycles of 10 s at 90 °C, 10 s at 58 °C, and 50 s at 72 °C. Thirdly, the AG1^1–450^ fragment was digested with *Eco*RI and *Kpn*I, while the AG1^450–917^ and AG1^917–1581^ fragments were digested with *Kpn*I/*Pst*I and *Pst*I/*Xho*I, respectively. Then, the digested products purified as described. The three purified DNA fragments were mixed with pET-15b-sumo vector, and the four DNA strands were sealed together at their sticky ends by DNA ligase to form the recombinant plasmid (pET-15b-sumo-AG1^1–1581^). The pET-15b-sumo-AG1^1–843^ and pET-15b-sumo-AG1^1–1581^ sequences were then identified by PCR, double enzyme digesting, and sequencing.

### 2.5 Protein expression and purification

All proteins were expressed in *E. coli* BL21(DE3) cells. Cells inoculated in 10 ml of LB containing 100 μg/ml of ampicillin. Cultures were grown by shaking at 200 rpm at 37 °C until the absorbance at 600 nm (A600) was ~ 1.0. This starter culture was then inoculated into 1.5 L of the same LB medium and grown as above until A600 = 0.8–1. Then, 0.3 mM IPTG was added, and incubation was continued for 18 h at 18 °C. Cells were then pelleted by centrifugation and re-suspended in lysis buffer (20 mM Tris–HCl, pH 7.5, 1000 mM NaCl, 10% glycerol (v/v)). The cells were sonicated and then centrifuged at 12,000 rpm for 30 min. The samples were loaded on to a Ni^2+^-charged IMAC column (GE Healthcare), bound with 120 ml of lysis buffer, and washed with 240 ml of washing buffer (20 mM Tris–HCl, pH 7.5, 300 mM NaCl, 10% glycerol (v/v), 50 mM imidazole). Then, the protein was eluted from the Ni^2+^ affinity column with elution buffer (20 mM Tris–HCl, pH 7.5, 300 mM NaCl, 10% glycerol (v/v), 500 mM imidazole). The eluted protein was incubated with SUMO protease (Invitrogen, Beijing) for 5 h at 18 °C to yield the mature proteins with two extra N-terminal amino acid residues (GluPhe). Protein was subsequently loaded on to a High Q Sepharose 6 Fast Flow column (GE Healthcare) and eluted with a 300–1000 mM NaCl gradient in buffer H (20 mM Tris–HCl, pH 7.5, 10% glycerol, 2 mM dithiothreitol (DTT)). The eluted fraction containing AG1^1–843^ or AG1^1–1581^ was collected, concentrated. The final purified protein was dialyzed against the storage buffer (20 mM Tris–HCl, pH7.5, 300 mM NaCl, 10% glycerol (v/v), 2 mM DTT) and was stored at − 80 °C until use.

### 2.6 Dynamic light scattering (DLS)

DLS measurements were performed at 25 °C with a DynaPro NanoStar instrument (Wyatt Technology Corporation) with a 20 μl micro-cuvette and a thermostat cell holder. The scattered light was collected at an angle of 90°. All buffers were filtered using a 0.22 μm filter membrane, and the samples were centrifuged (13,000*g* for 30 min at 4 °C). The measurement recording times ranged from 3 to 5 min (averaging 20–30 cycles every 10 s), and the data were analyzed with Dynamics 7.0 software using regularization arithmetic calculations (Wyatt Technology Corporation). The molecular weight (*Mw)* was calculated from the hydrodynamic radius using the empirical formula: *Mw* = (1.68 * *R*)^2.34^, where *R* is the hydrodynamic radius (in nm) and *Mw* is the molecular weight (in kDa). The protein concentrations used were 5, 10, 15, and 20 μM in buffer (300 mM NaCl, 20 mM Tris–HCl, pH7.5, 5% glycerol (v/v), 2 mM DTT), with a total sample volume of 50 μl.

### 2.7 Size exclusion chromatography (SEC)

SEC was carried out at constant temperature room (18 °C) using a fast protein liquid chromatography (FPLC) system (ÄKTA Purifier, GE Healthcare) on an analytical grade 24 ml Superdex 200 10/30 GL column (GE Healthcare). The same buffers were used as described in the DLS methods above. Fractions (0.3 ml) were collected at a flow of 0.3 ml/min, and the absorbance was surveyed at 260 and 280 nm. Experimental method is showed Shi et al. [2].

## Figures and Tables

**Fig. S1:**
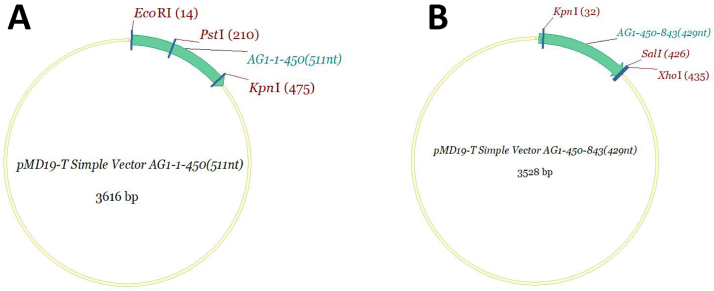
The pMD19-T simple vector containing AG1^1–450^ (A) and AG1^450–843^ (B).
