# Supplementary material for: Construction, expression, and characterization of AG11–843 and AG11–1581
Source: Data Brief. 2018 Aug 30;20:805–11. doi: 10.1016/j.dib.2018.08.094 (PMC6134160; doi:10.1016/j.dib.2018.08.094)
Supplement: Supplementary file 1 — Supplementary material [file mmc1.docx]

Conflicts of interest

There are no conflicts of interest to declare.
